# Molecular detection and phylogenetic characterization of pathogenic and endosymbiont microorganisms in *Hyalomma* ticks collected from livestock

**DOI:** 10.1186/s13071-026-07318-z

**Published:** 2026-03-11

**Authors:** Nighat Perveen, Daniil Iliashevich, Khalid Muhammad, Mourad Ben Said, Naganeeswaran Sudalaimuthuasari, Khaja Mohteshamuddin, Adnan Aldarwich, Olivier Andre Sparagano, Uday Kishore, Arve Lee Willingham

**Affiliations:** 1 Department of Biology, College of Science, United Arab Emirates University, P.O. Box 15551, Al-Ain, UAE; 2 Department of Veterinary Medicine, College of Agriculture and Veterinary Medicine, United Arab Emirates University, P.O. Box 15551, Al-Ain, UAE; 3 Zayed Center for Health Sciences, United Arab Emirates University, Al-Ain, UAE; 4 Laboratory of Parasitology, National School of Veterinary Medicine of Sidi Thabet, University of Manouba, 2010 Manouba, Tunisia; 5 Department of Basic Sciences, Higher Institute of Biotechnology of Sidi Thabet, University of Manouba, 2010 Manouba, Tunisia; 6 Khalifa Center for Genetic Engineering and Biotechnology, United Arab Emirates University, P.O. Box 15551, Al Ain, UAE; 7 Department of Infectious Diseases and Public Health, Jockey Club College of Veterinary Medicine and Life Sciences, City University of Hong Kong, Kowloon, Hong Kong SAR China

**Keywords:** *Hyalomma* ticks, Endosymbionts, Tick-borne pathogens, Phylogenetic analysis, Livestock, United Arab Emirates

## Abstract

**Background:**

*Hyalomma* ticks serve as vectors for a range of pathogens and harbor endosymbiotic bacteria that influence tick biology and the dynamics of pathogen transmission. In the United Arab Emirates, *Hyalomma dromedarii* and *H. anatolicum* are prevalent on livestock; yet, limited data exist on their microbial communities. This study aimed to determine the prevalence and phylogenetic relationships of key bacterial endosymbionts and tick-borne pathogens in *Hyalomma* ticks collected from livestock across all seven Emirates.

**Methods:**

A total of 671 ticks (532 *H. dromedarii* and 139 *H. anatolicum*) were collected from camels, cattle, sheep, and goats between October 2022 and October 2023. DNA was extracted and screened using specific polymerase chain reaction (PCR) assays targeting *Francisella*-like endosymbionts, ‘*Candidatus* Midichloria mitochondrii,’ Anaplasmataceae (‘*Candidatus* Neoehrlichia sp.’, uncultured *Ehrlichia* sp.), *Theileria*/*Babesia*, and *Trypanosoma brucei*. Representative amplicons were sequenced and phylogenetically analyzed.

**Results:**

In *H. anatolicum* collected from cattle, the following microorganisms were detected: ‘*Ca.* M. mitochondrii’ (54.2%; 95% confidence interval [CI] 45.2–63.0), *Francisella*-like endosymbionts (1.7%; 95% CI 0.2–5.9), uncultured *Ehrlichia* sp. (6.7%; CI 3.0–12.8), and *Theileria annulata* (5.0%; CI 1.8–10.6). In contrast, *H. dromedarii* collected from camels harbored only ‘*Ca.* M. mitochondrii’ (0.6%; 95% CI 0.1–1.8), and *Francisella*-like endosymbionts (7.1%; 95% CI 5.1–9.7). ‘*Candidatus* Neoehrlichia sp.’ (9.1%; CI 0.3–41.3) was detected exclusively in *H. anatolicum* ticks collected from sheep. In addition, *H. anatolicum* ticks collected from goats were positive for endosymbionts, with a high prevalence of ‘*Ca.* M. mitochondrii’ and *Francisella*-like endosymbionts (50.0%; 95% CI 15.7–84.3). *Trypanosoma brucei* was not detected. Co-infections occurred in 2.1% of ticks, predominantly involving both endosymbionts. Phylogenetic analyses revealed host-specific clustering patterns, with camel-derived sequences forming distinct clades from the cattle/sheep/goat-derived isolates for most taxa.

**Conclusions:**

This study provides the first comprehensive molecular survey of *Hyalomma*-associated microorganisms in the UAE, revealing high endosymbiont prevalence and significant host specificity in microbial communities. The detection of *T. annulata* exclusively in ticks collected from cattle, along with the absence of *T. brucei*, provides important insights into regional surveillance and control strategies. These findings enhance our understanding of tick–microbe interactions in an arid environment and support targeted vector control approaches.

**Graphical Abstract:**

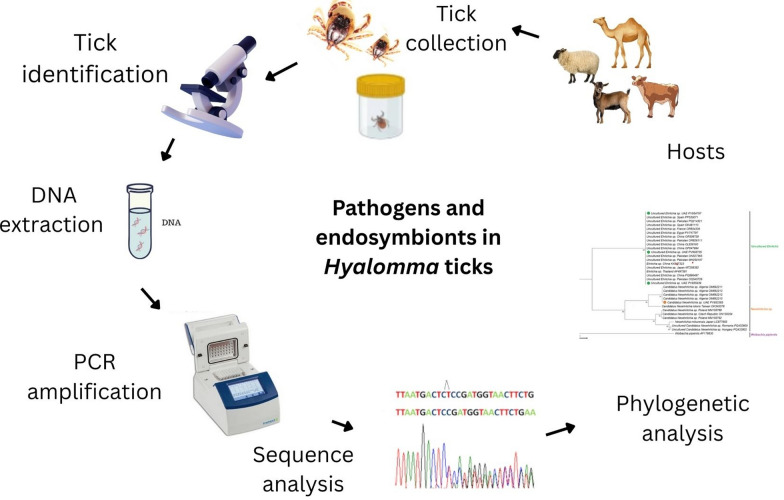

**Supplementary Information:**

The online version contains supplementary material available at 10.1186/s13071-026-07318-z.

## Background

Ticks are obligatory hematophagous arachnids that harbor a range of microorganisms, including pathogenic, commensal, and symbiotic species. Tick-borne pathogens (TBPs) comprise a diverse range of viruses, bacteria, and parasites responsible for numerous tick-borne diseases (TBDs) affecting humans and animals [1]. The role of tick endosymbionts in tick physiology and ecology is still not well understood. However, these endosymbionts significantly affect ticks as well as tick–pathogen interactions, which are key to determining disease risk. They can influence tick physiology by impacting nutritional adaptation and fitness and host immunity. Moreover, endosymbionts may shape disease ecology by interacting with TBPs, potentially facilitating or competing with pathogen development within the vector’s tissues [2].

Ticks are hematophagous parasites infesting a wide range of animals across the globe [3]. They pose a substantial threat to human and animal populations, since ticks are notoriously known for their ability to transmit TBPs, viruses, bacteria, and protozoans, causing morbidity and mortality in humans and animals in almost every part of the world [4]. For instance, tick-borne encephalitis virus, Alkhumra hemorrhagic fever (AHF), Crimean–Congo hemorrhagic fever virus (CCHFV), *Borrelia* spp., *Ehrlichia* spp., *Anaplasma* spp., *Francisella* spp., *Rickettsia* spp., and *Babesia* spp., as well as other pathogenic microorganisms, can cause deadly diseases if untreated or prevented effectively [4–6]. The abundance of tick species is known to successfully transmit infective agents to humans and animals, including those belonging to the following genera: *Ixodes* spp., *Haemaphysalis* spp., *Rhipicephalus* spp., *Dermacentor* spp., *Amblyomma* spp., and *Hyalomma* spp. [6, 7]. In recent years, there has been a growing interest in studying tick endosymbionts because they can shed light on various aspects of tick biology and ecology, along with the infective potential of TBPs, which are crucial for overcoming existing and emerging TBDs [2].

‘*Candidatus* Midichloria mitochondrii’ is an endosymbiotic intracellular α-proteobacterium that belongs to the order Rickettsiales [8]. Along with some mutualistic bacteria from the genera *Wolbachia*, *Coxiella*, *Rickettsia*, *Neorickettsia*, and *Francisella*, the current state of knowledge suggests that it does not cause any harm to the ticks but instead helps them to thrive, accelerating certain physiological processes (e.g., nutritional supplementation, especially B-vitamins and amino acids) [2, 9]. Originally found in *Ixodes ricinus*, ‘*Ca.* M. mitochondrii’ has more recently been described in other tick species [8, 10]. It predominantly resides in mitochondria of ticks’ oocytes, quite a unique characteristic. However, the exact role of ‘*Ca.* M. mitochondrii’ in the symbiotic relationship is still unclear [8, 11, 12].

The Anaplasmataceae family includes the genera *Ehrlichia*, *Anaplasma*, *Neorickettsia*, and *Neoehrlichia* [13]. These genera contain several pathogenic species such as *Ehrlichia canis*, *E. ewingii*, *E. chaffeensis*, *E. muris*, *E. ruminantium*, *Anaplasma phagocytophilum*, *A. marginale*, *A. centrale*, *A. ovis*, *A. bovis*, *A. platys*, and ‘*Candidatus* Neoehrlichia mikurensis’. They cause infections of medical and veterinary importance, including human granulocytic anaplasmosis, human monocytic ehrlichiosis, canine ehrlichiosis, and ovine anaplasmosis [13–16, 72]. These diseases are well documented and remain a significant concern in regions where ticks serve as vectors. They often present as nonspecific febrile illnesses, which contribute to frequent underdiagnosis and misdiagnosis. As a result, they are widely regarded as neglected conditions. [17, 18].

*Francisella* spp. includes a causative agent of tularemia, *Francisella tularensis*. This zoonotic disease can be transmitted via several routes, including arthropod-borne transmission through ticks. Tularemia is highly infectious and must be taken exceptionally seriously, as bacteria can be used as a bioweapon [19]. *Francisella*-like endosymbionts (FLEs) are closely related to the *Francisella* genus but are not known to cause disease in their hosts. As with other endosymbionts, they are thought to influence tick fitness, although the exact mechanisms remain unknown [20, 21].

Theileriosis is caused by haemoprotozoan parasites belonging to the genus *Theileria* spp. (Pirolasmorida: Theileriidae). The infection is of veterinary importance; no human cases have been reported [22]. *T. annulata*, *T. parva*, *T. equi*, *T. orientalis*, *T. lestoquardi*, *T. capreoli*, *T. ovis*, and *T. sergenti* are some of the pathogens that can be dangerous to both domestic and wild animals, ruminants in particular [22, 23]. *Theileria annulata*, the causative agent of tropical theileriosis, is endemic in Europe, Asia, the Middle East, and Africa. In susceptible cattle, infection induces marked lymphadenopathy, pyrexia, and extensive leukocyte infiltration of tissues. Progressive erythrocyte destruction and immune dysregulation lead to anemia, jaundice, and multiorgan dysfunction, contributing to the high mortality observed in untreated cases [1, 22]. Trypanosomes are also protozoan parasites transmitted primarily by specific haematophagous vectors, such as tsetse flies for salivarian species and certain biting flies for mechanically transmitted species, including *Trypanosoma evansi*. Ticks have been reported to carry trypanosomes’ DNA, including *T. evansi* and *Trypanosoma brucei,* in field surveys conducted in Egypt [24]. In the United Arab Emirates, these trypanosomes have been detected during investigations of vector-borne pathogens in dromedary camels [25]. However, the role of ticks as biological or mechanical vectors of *Trypanosoma* species remains unresolved.

*Hyalomma* ticks in the UAE carry a range of microbes, including endosymbionts [26–28]. Here, we assessed the molecular prevalence and diversity of key bacterial endosymbionts and TBPs in *H. dromedarii* and *H. anatolicum* ticks collected from major livestock hosts across all seven Emirates of the UAE. In this study, we have (1) investigated the occurrence of *Francisella*-like endosymbionts, ‘‘*Ca*. M. mitochondrii’,’ Anaplasmataceae members (including ‘*Ca*. Neoehrlichia sp.’ and uncultured *Ehrlichia* sp.), *Theileria*/*Babesia* piroplasmids, and *Trypanosoma brucei*; (2) determined overall and host-specific infection rates, and (3) performed phylogenetic analyses to elucidate the evolutionary relationships of the UAE isolates with global reference strains. These data will support targeted surveillance and control strategies for TBPs in the region.

## Methods

### Ethics statement and study design

This cross-sectional study was conducted in accordance with the experimental protocol approved by the Animal Research Ethics Committee of the United Arab Emirates University (ethical approval no. ERA-2022-1647). The study was designed to investigate the prevalence of TBPs and endosymbionts in *Hyalomma* ticks infesting livestock across all seven Emirates of the United Arab Emirates. The research followed a comprehensive sampling strategy to ensure representative coverage of different host species, geographical locations, and environmental settings. The study period extended between October 2022 and October 2023, providing a full annual cycle to assess pathogen prevalence. A multistage stratified sampling approach was employed to ensure adequate representation across the Emirates, host species, and farm types. The target sample size was calculated on the basis of an expected prevalence of 10% for major TBPs, with a 95% confidence level and 5% precision, resulting in a minimum required sample of 139 ticks per major host category, following the formula.

$${\mathrm{n}}\, = \,{\mathrm{Z}}^{{2}} \, \times \,{\mathrm{P}}\, \times \,{{\left( {{1} - {\mathrm{P}}} \right)} \mathord{\left/ {\vphantom {{\left( {{1} - {\mathrm{P}}} \right)} {{\mathrm{d}}^{{2}} }}} \right. \kern-0pt} {{\mathrm{d}}^{{2}} }}$$

n = required sample size.

*Z* = *z*-score corresponding to confidence level (1.96 for 95%).

*P* = expected prevalence (from prior studies 10%).

*d* = desired precision (margin of error 5%).

### Sampling sites and geographic coverage

Tick collection was performed across 46 sites distributed throughout all seven Emirates of the United Arab Emirates: Abu Dhabi, Dubai, Sharjah, Ajman, Fujairah, Umm Al Quwain, and Ras Al Khaimah. The sampling locations encompassed diverse environmental settings, including desert farms, commercial abattoirs, livestock markets, and zoological facilities, to capture the full spectrum of tick–host interactions in different ecological contexts. Site selection followed a randomized sampling design while maintaining farm confidentiality. Sites were distributed across different geographic zones according to the area of each Emirate.

### Animal selection and tick collection

A total of 671 tick specimens were collected from four livestock species: dromedary camels (*n* = 532, 79.3%), cattle (*n* = 120, 17.9%), sheep (*n* = 11, 1.6%), and goats (*n* = 8, 1.2%). Animal selection at each site was conducted using randomized sampling. For each selected animal, a standardized collection protocol was followed. Ticks were carefully removed using sterilized forceps, minimizing specimen damage and preventing cross-contamination between animals. Ticks’ preferred attachment sites included the perianal and vulvar regions, the inner thigh, udder, and inguinal regions [29]. Each tick was individually labeled with a unique identifier linking it to host species, collection site, date, and animal characteristics. Following collection, ticks were immediately placed in sterile collection tubes and stored in a portable ice box at 4 °C during field transportation. Specimens were subsequently transferred to the Parasitology and Entomology Laboratory at the Department of Veterinary Medicine, United Arab Emirates University. Upon arrival, ticks were stored at −20 °C until further processing.

### Quality control and sample management

To ensure sample integrity and prevent contamination, strict quality control measures were implemented throughout the collection process. Field collection equipment was sterilized between animals. A comprehensive sample database was maintained, including unique sample identifiers, collection date, location, host species, and sex. Sample tracking was ensured from field collection through the final molecular analysis.

### Morphological identification of ticks

All collected tick specimens were morphologically identified to the species level using established taxonomic keys and diagnostic criteria. The identification protocol was based on the standardized morphological characteristics described by Apanaskevich et al. [30] for *H.* (*Euhyalomma*) *dromedarii* and *H.* (*E*.) *schulzei*, and Walker et al. [31] for African tick species, including *H. anatolicum*. Identification focused on key morphological features, including capitular length, basis capituli morphology, palpal article structure, genital aperture characteristics, and ornamentation patterns. Quality assurance protocols were implemented to ensure accurate species identification. Representative specimens from each collection site and host species were preserved as voucher specimens in 70% ethanol.

### DNA extraction from ticks

All male ticks were subjected to DNA extraction. Prior to the extraction, each tick was surface-sterilized using 500 μL of 70% ethanol for 10 min, followed by washing with 500 μL sterile double-distilled water for 10 min to remove environmental contaminants [32]. Ticks were air-dried on the bench for 10 min, then manually homogenized using a plastic pellet pestle (Axygen, Corning, USA) in sterile 1.5 mL microcentrifuge tubes with liquid nitrogen. DNA extraction was performed using the DNeasy Blood and Tissue Kit (Qiagen, Hilden, Germany) according to the manufacturer’s protocol. DNA elutes were stored at −20 °C until further use.

### Polymerase chain reaction and agarose gel electrophoresis

Polymerase chain reaction (PCR) screening targeted *Francisella* spp., Anaplasmataceae family bacteria, *Theileria*/*Babesia* spp., and *T. brucei* using specific primer sets. *Francisella* detection employed primers amplifying a 1,151-base pair (bp) fragment of the 16S rRNA gene [33]. Anaplasmataceae members were detected using primers targeting a 345-bp portion of the 16S rRNA gene [34]. *Theileria* and *Babesia* spp. were identified using primers designed for a 560-bp fragment of the ssrRNA gene [35], while *T. brucei* detection utilized primers targeting a 164-bp trypanosomal-specific DNA sequence [24]. Each 25-μL PCR reaction contained 4 μL FIREPol Master Mix (Solis BioDyne, Tartu, Estonia), 1.0 μL (10 pM) of each primer, 2 μL genomic DNA, and 17 μL nuclease-free water. Amplifications were performed in a TC 9639 thermal cycler (Benchmark Scientific, USA) under the following conditions: *Francisella* (95 °C/4 min; 40 cycles: 95 °C/30 s, 60 °C/45 s, 72 °C/60 s; 72 °C/20 min); Anaplasmataceae (95 °C/2 min; 40 cycles: 94 °C/1 min, 54 °C/30 s, 72 °C/30 s; 72 °C/5 min); *Theileria*/*Babesia* (94 °C/2 min; 35 cycles: 94 °C/30 s, 50 °C/30 s, 72 °C/60 s; 72 °C/7 min); and *T. brucei* (95 °C/3 min; 33 cycles: 94 °C/30 s, 52 °C/30 s, 72 °C/30 s; 72 °C/10 min). Complete primer details are provided in Additional file 1: Table S1. Negative controls (nuclease-free water) and positive controls were included to monitor contamination and primer functionality. Master mix preparation and template loading were performed in separate laboratories to prevent cross-contamination. PCR products were visualized on 1.5% agarose gels with RedSafe^™^ nucleic acid staining solution (iNtRON Biotechnology, Republic of Korea) using a NuGenius Gel Documentation Workstation (Syngene, UK). Positive amplicons were preserved for DNA sequencing.

### Sequencing and phylogenetic analysis

Sanger sequencing was conducted on the positive samples at the Biology Department sequencing unit, United Arab Emirates University. Amplicon sequencing chromatograms were analyzed and edited using SnapGene (snapgene_8.1.1_win.exe) tool. Further, amplicon sequences were compared against National Center for Biotechnology Information (NCBI) databases using BLASTn (https://blast.ncbi.nlm.nih.gov/Blast.cgi, accessed July 15, 2025) for taxonomic identification. Moreover, homologous sequences with 99–100% query coverage were downloaded from the NCBI database for phylogenetic analyses. Multiple sequence alignments were generated using MUSCLE with default parameters, followed by manual trimming. Optimal substitution models were selected on the basis of the lowest Bayesian information criterion (BIC) scores. Neighbor-joining trees were constructed using the Kimura 2-parameter method with 1000 bootstrap replicates in MEGA 11 (v11.0.13) [36]. Amplicon sequences were submitted to GenBank via the BankIt submission portal (https://www.ncbi.nlm.nih.gov/WebSub/?tool=genbank) and obtained the following accession nos: *Francisella* sp. (PV959455, PV959325, PV958095, PV956212, PV954827, and PV954819); ‘*Ca.* Neoehrlichia sp.’ (PV953365); uncultured *Ehrlichia* sp. (PV955939, PV955725, and PV954797); ‘*Ca.* M. mitochondrii’ (PV952972, PV952993, PV952994, PV956136, and PV956164); and *T. annulata* (PV954755 and PV955656). Infection rates were calculated on the basis of the number of positive samples per host species.

### Data and statistical analysis

Prevalence of each pathogen and endosymbiont was calculated as the proportion of positive individuals relative to the total number of ticks tested, with 95% confidence intervals estimated using the Clopper–Pearson method. Differences in pathogens’ prevalence among tick species were assessed using chi-squared or Fisher’s exact tests, as appropriate. A mixed-effects logistic regression model was constructed in R v4.2.2 (R Foundation for Statistical Computing, Vienna, Austria), with tick infection status as the binary outcome and fixed effects for host species; tick species was included as a random intercept to account for clustering. Model selection was based on Akaike’s information criterion, and goodness-of-fit was assessed using the Hosmer–Lemeshow test. Co-infection patterns were explored using pairwise association odds ratios. All statistical analyses were performed in R, with significance set at *P* < 0.05.

## Results

### Tick identification

All specimens were confirmed as ixodid ticks through the examination of definitive morphological features including the shape of the basis capituli, palp segmentation, scutum ornamentation, and genital aperture structure. Ticks collected from camels exhibited the characteristic elongate mouthparts, punctate scutum, and lateral palpal projections diagnostic of *H. dromedarii*. Conversely, ticks collected from cattle, sheep, and goats displayed the narrower palps, smoother scutum margins, and distinct adanal plates diagnostic of *H. anatolicum*.

### Overall prevalence of pathogens and endosymbionts

A total of 671 tick specimens, comprising 532 *H. dromedarii* and 139 *H. anatolicum*, were screened by PCR for five targets. Tick-borne microbes were detected in 136 out of 671 DNA samples extracted from ticks, with an overall infection rate of 20.3%. *Francisella*-like endosymbionts were detected in 45/671 specimens, yielding an overall prevalence of 6.7% (95% CI 5.0–8.8). ‘‘*Ca*. M. mitochondrii’’ was identified in 76/671 ticks (11.3%; 95% CI 9.1–13.9) (Table 1). *T. annulata* DNA was amplified in 6/671 specimens (0.8%; 95% CI 0.4–1.9), while uncultured *Ehrlichia* sp. and ‘*Ca*. Neoehrlichia sp.’ occurred at 1.1% (95% CI 0.6–2.3) and 0.1% (95% CI 0.0–0.8), respectively. *T. brucei* was not detected in any individual sample. Chi-squared test of homogeneity revealed significant differences in prevalence among the five pathogens and endosymbionts (*χ*^2^ = 161.0, *df* = 4, *P* < 0.001), confirming heterogeneous distribution patterns across the targeted microorganisms. The highest infection rates were observed for endosymbionts (‘‘*Ca*. M. mitochondrii’’ and FLE) compared with pathogenic agents (*Theileria* and Anaplasmataceae), with ‘‘*Ca*. M. mitochondrii’’ showing significantly higher prevalence than FLE (76% versus 45%; *χ*^2^ = 8.1, *df* = 1, *P* < 0.001).* Theileria annulata* was detected only in *H. anatolicum* ticks collected from cattle in Sharjah, whereas ‘*Ca.* Neoehrlichia sp.’ was detected only in *H. anatolicum* ticks collected from sheep in Al Ain (Additional file 2: Table S2).

**Table 1:** Comparison of pathogen and endosymbiont prevalence in *Hyalomma* ticks

Pathogen/endosymbiont	Positive (*n*/*N*)	Prevalence (%)	95% CI (%)
‘*Ca.* M. mitochondrii’	76/671	11.3	9.1–13.9
*Francisella*-like endosymbionts	45/671	6.7	5.0–8.8
Uncultured *Ehrlichia* sp.	8/671	1.1	0.6–2.3
*T. annulata*	6/671	0.8	0.4–1.9
‘*Ca.* Neoehrlichia sp.’	1/671	0.1	0.0–0.8
*T. brucei*	0/671	0.0	0.0–0.5

Chi-squared comparisons between individual pathogen and endosymbiont prevalences demonstrated highly significant differences. *Francisella*-like endosymbionts were significantly more prevalent than ‘*Ca.* M. mitochondrii’ (*χ*^2^ = 8.1, *df* = 1, *P* < 0.01) and *T. annulata* (*χ*^2^ = 29.4, *df* = 1, *P* < 0.001). Similarly, ‘*Ca*. M. mitochondrii’ exhibited a higher prevalence than *T. annulata* (*χ*^2^ = 61.8, *df* = 1, *P* < 0.001). Comparisons between pathogenic taxa also revealed significant heterogeneity: *T. annulata* versus ‘*Ca*. Neoehrlichia sp.’ (*χ*^2^ = 2.3, *df* = 1, *P* = 0.13) and ‘*Ca*. Neoehrlichia sp.’ versus uncultured *Ehrlichia* sp. (*χ*^2^ = 4.0, *df* = 1, *P* = 0.04). Overall, the chi-squared test of homogeneity across all five targets confirmed a heterogeneous distribution pattern (*χ*^2^ = 161.0, *df* = 4, *P* < 0.001)

Overall, infection rates varied markedly among endosymbionts and pathogens (Table 2). *Hyalomma dromedarii* collected from camels tested positive for only two microorganisms. *Francisella*-like endosymbionts were detected in 38/532 specimens, yielding a prevalence of (7.1%; 95% CI 5.1–9.7) and ‘*Ca*. M. mitochondrii’ was identified in 3/532 ticks (0.65%; 95% CI 0.1–1.8). However, *H. anatolicum* ticks tested positive for all five microorganisms (Table 2). *Theileria annulata* DNA was amplified in 6/120 samples collected from cattle (5%; 95% CI 1.8–10.6), while ‘*Ca*. Neoehrlichia sp.’ was detected in 1/11 samples collected from sheep with a prevalence of 9.1% (95% CI 0.3–41.3), and uncultured *Ehrlichia* sp. occurred in 8/120 samples from cattle (6.7%; 95% CI 3.0–12.8).

**Table 2:** Infection rates of endosymbionts and pathogens in *Hyalomma* ticks collected from livestock in the UAE

Host	Tick species	*N* = samples	‘*Ca.* M. mitochondrii’ *n* (%) [95% CI]	*Francisella*-like endosymbionte *n* (%) [95% CI]	Uncultured *Ehrlichia* sp. *n* (%) [95% CI]	‘*Ca.* Neoehrlichia sp.’ *n* (%) [95% CI]	*T. annulata* *n* (%) [95% CI]
Camel	*H. dromedarii*	532	3 (0.6) [0.1–1.8]	38 (7.1) [5.1–9.7]	0 (0.0) [0.0–0.7]	0 (0.0) [0.0–0.7]	0 (0.0) [0.0–0.7]
Cattle	*H. anatolicum*	120	65 (54.2) [45.2–63.0]	2 (1.7) [0.2–5.9]	8 (6.7) [3.0–12.8]	0 (0.0) [0.0–2.5]	6 (5.0) [1.8–10.6]
Sheep	*H. anatolicum*	11	4 (36.4) [10.9–69.2]	1 (9.1) [0.3–41.3]	0 (0.0) [0.0–24.7]	1 (9.1) [0.3–41.3]	0 (0.0) [0.0–24.7]
Goat	*H. anatolicum*	8	4 (50.0) [15.7–84.3]	4 (50.0) [15.7–84.3]	0 (0.0) [0.0–36.9]	0 (0.0) [0.0–36.9]	0 (0.0) [0.0–36.9]
Total		671	76 (11.3)	45 (6.7)	8 (1.2)	1 (0.1)	6 (0.9)

A chi-squared test of homogeneity across host species and microbial targets revealed significant variation in infection rates among the five microorganisms (*χ*^2^ = 161.0, *df* = 4, *P* < 0.001)

### Co-infection patterns

Overall, co-infections were detected in 14 of 671 tick samples (2.1%; 95% CI 1.0–3.1), involving ‘*Ca*. M. mitochondrii’ and FLEs (7/671; 1.0%; 95% CI 0.2–1.8) in ticks collected from camel (Al Ain), cattle (Dubai and Sharjah), sheep (Dubai), and goats (Abu Dhabi), ‘*Ca*. M. mitochondrii’ and *T. annulata* (6/671; 0.8%; 95% CI 0.1–1.6) in ticks collected from cattle (Sharjah), and *Francisella*-like endosymbionts and uncultured *Ehrlichia* sp. (1/671; 0.1%; 95% CI −0.1 to 0.4) in ticks collected from cattle (Al Ain).

### Molecular detection and phylogenetic analysis of *Francisella*-like endosymbiont

**Fig. 1:**
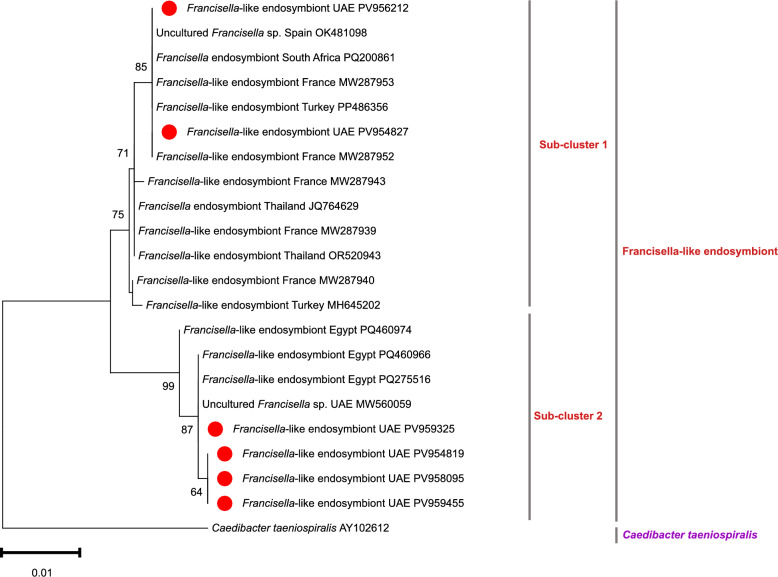
Phylogenetic tree of *Francisella*-like endosymbionts inferred from partial 16S rRNA sequences using the neighbor-joining method. The tree shows the placement of sequences isolated from *Hyalomma* ticks infesting livestock in the UAE. Bootstrap values from 1000 replicates (only those above 50%) are indicated at the nodes. Newly obtained sequences are marked with red circles. *Caedibacter taeniospiralis* was included as the outgroup. The tree was constructed using MEGA-11 [36]

*Francisella* sp. DNA was amplified from 45 *Hyalomma* tick specimens (both *H. dromedarii* and *H. anatolicum*) collected from all livestock hosts across the seven Emirates. Sequencing of the 1151-bp 16S rRNA amplicons generated six unique GenBank accessions (PV959455, PV959325, PV958095, PV956212, PV954827, and PV954819), each exhibiting 99–100% identity with *Francisella*-like endosymbionts previously recorded in Egypt, France, and Turkey (Additional file 3: Table S3). Maximum likelihood reconstruction under the HKY + G substitution model (1000 ultrafast bootstraps) resolved all the UAE isolates into a strongly supported monophyletic clade (bootstrap = 98%) distinct from pathogenic *Francisella* species (Fig. 1). This clade comprised two well-defined subclusters. Subcluster 1 comprised *H. anatolicum*-derived sequences from cattle and goat ticks, clustering with European and African reference strains from France, Spain, South Africa, and Turkey (bootstrap = 87%). Subcluster 2 included sequences from both tick species but was dominated by *H. dromedarii*-derived isolates, which grouped tightly with Egyptian and earlier UAE sequences (bootstrap = 85%).

### Molecular detection and phylogenetic analysis of ‘*Candidatus* Neoehrlichia sp.’

**Fig. 2:**
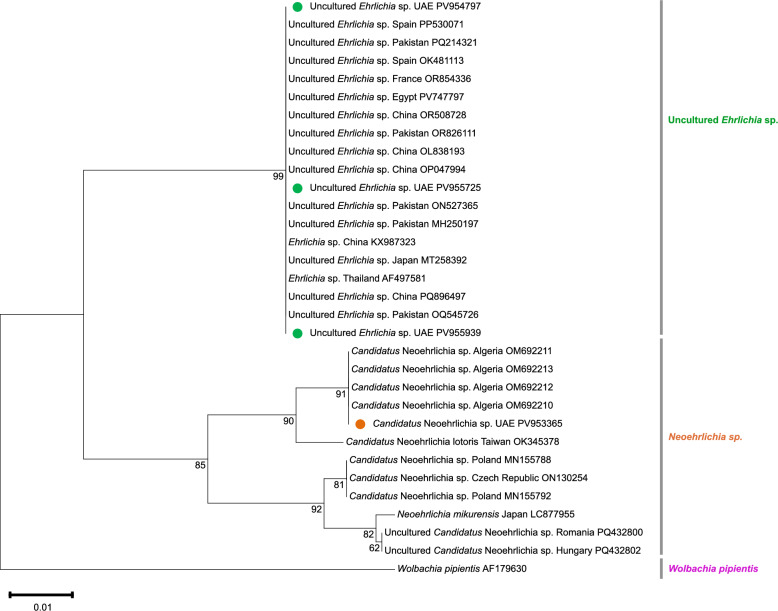
Phylogenetic tree inferred from partial 16S rRNA sequences using the neighbor-joining method, illustrating the relationships of ‘*Ca.* Neoehrlichia sp.’ and uncultured *Ehrlichia* sp. isolates obtained from *Hyalomma* ticks infesting livestock in the UAE. Bootstrap support values from 1000 replicates are indicated at the nodes (only values > 50% shown). Newly obtained uncultured *Ehrlichia* sp. sequences are marked with green circles, and the ‘*Ca.* Neoehrlichia sp.’ sequence is indicated with an orange circle. *Wolbachia pipientis* was used as the outgroup. The phylogeny was constructed using MEGA 11 software [36]

The 16S rRNA gene fragment of ‘*Ca.* Neoehrlichia sp.’ was identified on the basis of sequence similarity with GenBank records (Additional file 2: Table S2). ‘*Candidatus* Neoehrlichia sp.’ DNA was detected exclusively in *H. anatolicum* ticks collected from sheep in Al Ain, Abu Dhabi. The 345-bp fragment of 16S rRNA gene of ‘*Ca.* Neoehrlichia sp.’ was not detected in *H. dromedarii* or *H. anatolicum* from other hosts such as camels, cattle, and goats nor from other Emirates including Dubai, Sharjah, Ajman, Umm Al Quwain, Ras Al Khaimah, and Fujairah. The sequence obtained was deposited in GenBank under accession no. PV953365. This sequence showed 100% identity to ‘*Ca.* Neoehrlichia sp.’ previously reported from *Bovicola ovis* in Algeria (OM692212 and OM692210), and 99.62% similarity with OM692211, 99.24% with OM692213 from the same species in Algeria (Additional file 3: Table S3). In addition, it showed 98.47% similarity with ‘*Candidatus* Neoehrlichia lotoris’ detected from *Rhipicephalus sanguineus* collected from dogs in Taiwan. The phylogenetic tree (Fig. 2) constructed using related ‘*Ca.* Neoehrlichia sp.’ sequences from GenBank showed that the UAE isolate clustered closely with ‘*Ca.* Neoehrlichia spp.’ detected in Algeria.

### Molecular detection and phylogenetic analysis of uncultured *Ehrlichia* sp.

Uncultured *Ehrlichia* sp. DNA was detected exclusively in *H. anatolicum* ticks collected from cattle in the Emirates of Abu Dhabi, Dubai, and Sharjah. This 345-bp fragment of the 16S rRNA gene was absent in *H. dromedarii* and *H. anatolicum* collected from camels, sheep, and goats in Ajman, Umm Al Quwain, Ras Al Khaimah, and Fujairah. The sequences obtained were deposited under GenBank accession nos. PV955939, PV955725, and PV954797. These sequences shared 99–100% identity with uncultured *Ehrlichia* sp. from Pakistan, China, and Thailand (Additional file 3: Table S3), including a 100% match with uncultured *Ehrlichia* sp. ON527365 from ticks infesting sheep and goats in Pakistan, and *Ehrlichia* sp. AF497581 was isolated from *Rhipicephalus* (*Boophilus*) *microplus* in Thailand. Phylogenetic analysis placed these UAE isolates in a cluster with sequences from Pakistan, Spain, Egypt, France, and Japan, as well as with *Ehrlichia* sp. references from China (KX987323) and Thailand (AF497581) (Fig. 2).

### Molecular detection and phylogenetic analysis of ‘*Ca*. M. mitochondrii’

**Fig. 3:**
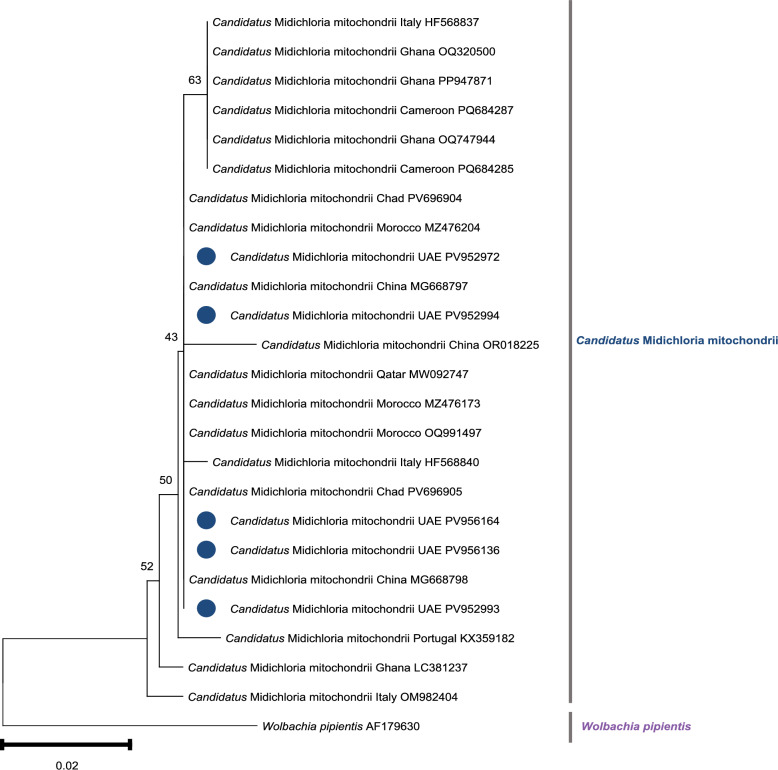
Phylogenetic tree of ‘*Ca.* M. mitochondrii’ inferred from partial 16S rRNA sequences using the neighbor-joining method. The tree shows the position of sequences isolated from *Hyalomma* ticks infesting UAE livestock. Bootstrap values from 1000 replicates (only those above 50%) are presented at nodes. Newly obtained sequences are marked with blue circles. *Wolbachia pipientis* was used as the outgroup. The tree was constructed using MEGA-11 software [36]

‘*Candidatus* M. mitochondrii’ DNA was detected by PCR in both *H. dromedarii* and *H. anatolicum* ticks collected from all host species across the Emirates of Abu Dhabi, Dubai, Sharjah, and Ajman. A total of 76 positive samples yielded sequences deposited in GenBank under accession nos. PV952972, PV952993, PV952994, PV956136, and PV956164. These sequences exhibited 100% identity with ‘*Ca.* M. mitochondrii’ (Additional file 3: Table S3) that was previously detected in *Hyalomma* ticks collected from camels in Chad (PV696904 and PV696905); *Hyalomma aegyptium* parasitizing *Testudo graeca* in Qatar (MW092747); *H. anatolicum* ticks in China (MG668797); and ticks from Morocco (MZ476204). Phylogenetic analysis showed that sequences from this study clustered closely with ‘*Ca.* M. mitochondrii’ sequences from Chad, Morocco, China, Qatar, and Italy (Fig. 3).

### Molecular detection and phylogenetic analysis of *Theileria annulata*

**Fig. 4:**
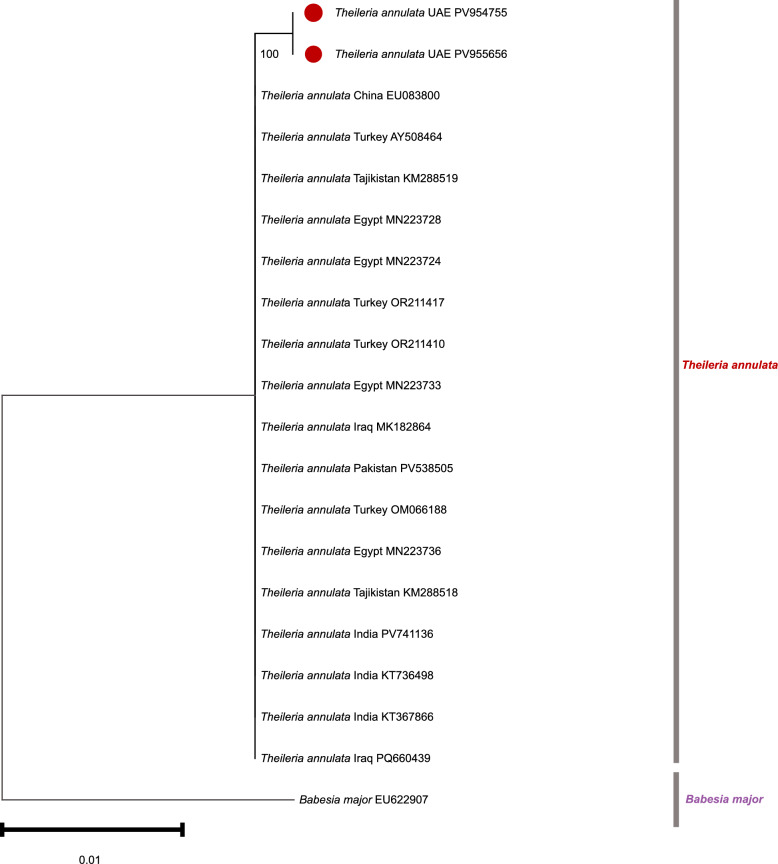
Phylogenetic tree of *T. annulata* inferred from partial 18S rRNA sequences using the neighbor-joining method. The tree shows the phylogenetic placement of sequences isolated from *Hyalomma* ticks infesting UAE livestock. Newly obtained sequences are marked with brown circles. Bootstrap percentages from 1000 replicates (only values above 50%) are shown at the nodes. *Babesia major* was used as the outgroup. The phylogeny was constructed using MEGA-11 software [36]

Nucleotide sequences of 18S rRNA gene PCR products from ticks were compared with GenBank records (Additional file 3: Table S3) and identified as *T. annulata*. *Theileria annulata* DNA was detected exclusively in *H. anatolicum* ticks collected from cattle in Sharjah. The sequences obtained in this study were submitted to the GenBank under accession nos. PV954755 and PV955656. Sequence similarity among *T. annulata* isolates ranged between 99.80% and 99.81% (Additional file 3: Table S3), closely matching *T. annulata* sequences reported from China (EU083800), Turkey (AY508464), Egypt (MN223728, MN223724, and MN223733), and Iraq (MK182864). Phylogenetic analysis (Fig. 4) revealed UAE sequences clustering with isolates from India, Egypt, Turkey, Iraq, and Pakistan. No piroplasmids were detected in *H. dromedarii* or *H. anatolicum* ticks collected from other hosts or Emirates.

## Discussion

This study examined the ticks collected from camels, cattle, sheep, and goats in the different Emirates of the UAE. The detection of pathogens and endosymbionts in these ticks suggests that they are likely to act as carriers of microorganisms and potentially transmit them. Such microorganisms can be acquired either vertically from the parent generation or horizontally during blood feeding at the larval or nymphal stages and subsequently maintained through transstadial transmission to adulthood. The specific hosts on which the ticks had fed during earlier stages of life could not be determined. Nevertheless, it is plausible that the collected ticks harbored parasites from multiple species. Notably, *Hyalomma* ticks are recognized for their broad host range [29]. Five different microorganisms were detected in the ticks in this study, including ‘*Ca.* M. mitochondrii,’ ‘*Ca.* Neoehrlichia sp.,’ *Francisella*-like endosymbiont, uncultured *Ehrlichia* sp., and *T. annulata*. The first three are known endosymbionts. In a previous study performed on DNA from *Hyalomma* ticks, *Francisella* sp., SFG *Rickettsia*, *T. annulata*, and *T. ovis* were detected [28]. These findings demonstrate the capacity of ticks to harbor a range of endosymbionts and pathogens, underscoring their role as significant vectors of infectious agents affecting both humans and animals.

This study reports the first molecular detection of ‘*Ca.* M. mitochondrii’ in *Hyalomma* ticks from the UAE. ‘*Ca.* M. mitochondrii’ is an intracellular α-proteobacterial symbiont that was previously detected in several hard tick (Ixodidae) species [37, 38]. The bacterium was found to be localized both in the cytoplasm and in the intermembrane space of the mitochondria of tick ovarian cells. It is the first bacterium shown to reside within the mitochondria [8, 38]. In this study, ‘*Ca.* M. mitochondrii’ was detected in both *Hyalomma* species, although with a high prevalence in *H. anatolicum* ticks collected from cattle (54.2%), sheep (36.4%), and goats (50%) and a low prevalence in *H. dromedarii* (0.6%). Previously, ‘*Ca.* M. mitochondrii’ has been reported in ticks infesting Tunisian dromedaries [39]. In particular, the infection by this bacterium was identified in *H. impeltatum* (10%) and *H. dromedarii* (6%) [39]. This bacterium has also been reported within many tick species, such as *I. ricinus* [8], *Rhipicephalus bursa* [38], *R. sanguineus* s.l. [40], and *Dermacentor andersoni* [41]. The possible role of this endosymbiont in the ticks is yet to be investigated.

*Francisella*-like endosymbionts are facultative intracellular bacteria that are prevalent in natural environments [2, 42]. The bacterium may be transmitted to animals and humans through tick bites [43], as it lives in the salivary glands [44, 45]. In addition, the bacterium may spread via transovarial and transstadial transmission in ticks. For example, it was detected in the reproductive tissues of female *D. andersoni* [46], and unfed infected larvae of *Amblyomma americanum* in nature, supporting the idea of transovarial transmission [47]. However, transstadial transmission has been confirmed under laboratory conditions for multiple tick species, including *D. andersoni*, *Dermacentor variabilis*, and *A. americanum* [48]. FLEs are found in human-biting ticks, including *Amblyomma*, *Dermacentor*, *Hyalomma*, and *Ixodes* [20, 28, 42, 49, 50]; however, the bacteria were found to be nonpathogenic to humans [51]. In the present study, tick-borne microbes were detected using PCR, and FLE was reported in *Hyalomma* ticks collected from all animals, with the highest prevalence (50%) in *H. anatolicum* ticks collected from goats; this is consistent with earlier studies where FLEs were reported with high prevalence in *Hyalomma* ticks (60–100%) [52, 53]. However, the genus *Francisella* was reported with high prevalence (72%) in *H. anatolicum* ticks in the UAE [27] and (68%) in Pakistan using 16S rRNA gene-based analysis [54]. FLEs are widespread in natural settings, and survival depends on sunlight, temperature, and other factors [55, 56], including their associations with various animals [42]. Therefore, the genus *Francisella* was detected with high relative abundance in *Hyalomma* ticks in all-natural samples, using 16S rRNA gene-based analysis studies in the UAE [26, 27, 57, 58], Saudi Arabia [59, 60], Iran [61], Pakistan [54, 62], Tunisia [63], and Kenya [64]. FLEs improve tick fitness by supplying vitamins and cofactors found in low concentrations in vertebrate blood; therefore, elimination of FLEs causes physical abnormalities and deficiencies in ticks [65, 66] that can be an effective tick control strategy.

The Anaplasmataceae family includes obligate, arthropod-transmitted intracellular bacteria that can be zoonotic and potentially fatal [67]. Ehrlichiosis in an emerging vector-borne rickettsial zoonotic disease of worldwide distribution. *Ehrlichia* species are tick-transmitted Gram-negative obligate intracellular bacteria that infect mature and immature hematopoietic cells [68]. It causes an infectious disease of mammals owing to different species of the genus *Ehrlichia* [68]. Currently, the genus *Ehrlichia* includes the species *E. canis*, *E. chaffeensis*, *E. muris*, *E. ewingii*, *E. ruminantium*, and *E. minasensis* [69, 70], which are responsible for emerging zoonoses in humans [71–73]. To our knowledge, this is the first report of ‘*Ca.* Neoehrlichia sp.’ in *H. anatolicum* ticks collected from sheep in the UAE. The bacterium was found with a prevalence of 9% in the present study and is closely related to ‘*Candidatus* Neoehrlichia lotoris’ [74, 75]. This species has previously been detected in an Austrian fox [74], a red fox in the Czech Republic [76], a raccoon dog in Central Europe [77], a European badger, and *Ixodes* ticks [78]. In addition, ‘*Ca.* Neoehrlichia mikurensis’ has been detected in *I. ricinus* ticks in Central Europe (4.5%) [79]. Human infections are reported in China, where patients have been exposed to areas with high rates of tick activity [80]. Uncultured *Ehrlichia* sp. (6.7%) has been detected in *H. anatolicum* collected from cattle. The results are consistent with the study conducted in Pakistan exploring the genetic diversity of TBPs in livestock, which detected *Ehrlichia* sp. in *H. anatolicum* ticks [81]. Detected microorganisms, such as uncultured *Ehrlichia* and Neoehrlichia species, are of zoonotic concern and need to be investigated for their prevalence, transmission dynamics, and clinical relevance in humans, livestock, and wildlife. These findings can prompt integrated One Health studies that include human, animal, and environmental health professionals to assess public health risks.

*Theileria* species infect a wide range of domestic and wild animals and are transmitted by a variety of ixodid ticks belonging to the genera *Amblyomma*, *Haemaphysalis*, *Hyalomma*, and *Rhipicephalus* [22]. Many of these tick species are associated with substantial economic losses in the livestock industry, primarily owing to disease transmission, increased mortalities, hide damage, and reduced productivity in domestic animals. [1]. Theileriosis causes major economic losses in the livestock sector worldwide [82]. Immunization with a live attenuated vaccine is an effective prophylactic measure. Several *Theileria* species have been reported across the Middle East and North Africa (MENA) region [1]. In this study, we found *T. annulata* with low prevalence (5%) in Sharjah, only consistent with our previous study results from the UAE [28]. However, Ismaeil et al. [83] reported a high prevalence of *Theileria* species (52.7%) from cows using microscopy in Sharjah, UAE. The variation in *Theileria* species detection across the UAE may be attributed to differences in environmental factors (such as temperature and humidity), livestock farming practices (including animal movement and imported animals), local tick burdens, livestock breeds, host susceptibility, frequency of acaricide application, geographic distribution of *Hyalomma* ticks (the vector), variations in veterinary surveillance, and diagnostic methods across the different Emirates. The genotype clustered with *T. annulata* identified from India, China, Turkey, Egypt, Iraq, and Pakistan, suggesting that the current study genotype could be a geographically widespread variant. The prevalence of *Theileria* in livestock from Oman [84] and Saudi Arabia [85] is also comparable to our findings. Furthermore, the highest prevalence of *Theileria* infections occurs in *H. anatolicum* compared with other species, suggesting that *H. anatolicum* may be the main vector of theileriosis [86]. In one previous study, species-wise prevalence analysis revealed a significantly high prevalence rate of *T. annulata* in *H. anatolicum* ticks in comparison with *R.* (*B.*) *microplus*, transmitting the pathogen to the bovine population [87]. The present findings, together with reports from other studies in the region, suggest that the Arabian Peninsula could represent an area where theileriosis may become endemic in the future. However, the role of acaricide application should be carefully considered, and future studies should investigate livestock populations treated with and without acaricides to assess their potential as risk factors for theileriosis.

*Trypanosoma* spp. DNA was not detected in the tick samples analyzed in this study, in contrast to some previous reports [24, 88]. Although ticks have occasionally been reported to harbor Trypanosomatidae DNA in field surveys, such detections are generally interpreted as incidental and possibly related to residual host blood rather than evidence of vector competence. The absence of *Trypanosoma* DNA in the present study is therefore consistent with the current understanding that ticks are unlikely to play a significant role in trypanosome transmission in the tsetse-free environment of the United Arab Emirates.

Our study had some limitations. First, only adult male ticks were included in the molecular analyses, as female ticks were either underrepresented or excluded owing to engorgement, which can complicate accurate morphological identification and introduce host-derived DNA that may confound pathogen detection. Second, although we intended to screen for both *T. evansi* and *T. brucei*, previously reported in ticks, the *T. evansi*-specific primers did not produce reliable amplification under our experimental conditions; therefore, only *T. brucei* results were reported. Third, the absence of *Trypanosoma* spp. in tick samples does not exclude their presence in vertebrate hosts, as trypanosomes are primarily blood-borne parasites transmitted by other hematophagous vectors, and detection in ticks, when reported, may reflect incidental carriage. Future studies incorporating multiple tick life stages, engorgement statuses, and a broader range of pathogens will help to better clarify the epidemiological role of ticks in pathogen transmission in the region.

## Conclusions

This study provides molecular evidence of diverse range of tick-associated microorganisms in *Hyalomma* ticks from the UAE. Notably, to our knowledge, it reports the first detection of ‘*Ca*. M. mitochondrii’, ‘*Ca*. Neoehrlichia sp.,’ and an uncultured *Ehrlichia* sp. in *Hyalomma* ticks from the UAE, highlighting the need for further investigations to determine their biological roles and potential relevance to animal and public health. Furthermore, the absence of *Trypanosoma* spp. DNA supports the view that ticks are unlikely to play a significant role in trypanosome transmission in this tsetse-free region.

## Supplementary Information

Additional File 1: Table S1. Primer details for molecular identification of microorganisms. Table S2. Microorganisms detected from ticks, host details and tick collection sites. Table S3. Query sequence similarities with GenBank subject sequences.

## Data Availability

All sequence data generated in this study have been deposited in GenBank under accession nos. PV959455, PV959325, PV958095, PV956212, PV954827, PV954819, PV953365, PV955939, PV955725, PV954797, PV952972, PV952993, PV952994, PV956136, PV956164, PV954755, and PV955656.

## Abbreviations

TBPs: Tick-borne pathogens
TBDs: Tick-borne diseases
AHF: Alkhumra hemorrhagic fever
CCHFV: Crimean–Congo hemorrhagic fever virus
UAE: United Arab Emirates
FLEs: *Francisella*-like endosymbionts
MENA: Middle East and North Africa
