# Supplementary material for: Molecular detection and phylogenetic characterization of pathogenic and endosymbiont microorganisms in Hyalomma ticks collected from livestock
Source: Parasit Vectors. 2026 Mar 11;19:171. doi: 10.1186/s13071-026-07318-z (PMC13094174; doi:10.1186/s13071-026-07318-z)
Supplement: Supplementary file 1 — Additional File 1: Table S1. Primer details for molecular identification of microorganisms. Table S2. Microorganisms detected from ticks, host details and tick collection sites. Table S3. Query sequence similarities with GenBank subject sequences. [file 13071_2026_7318_MOESM1_ESM.docx]

**Table S1.** Primer details for molecular identification of microorganisms.

| **Pathogen** | **Target gene** | **Primer** | **Sequence (5’-3’)** | **Cycle conditions** | **Amplicon size** | **Reference** |
| --- | --- | --- | --- | --- | --- | --- |
| *Francisella*  spp. | 16S rRNA | Fr153F0.1 | GCCCATTTGAGGGGGATACC | 95°C 4 min  40 cycles:  94°C 30 s  60°C 45 s  72°C 60 s  72°C 20 min | 1151 bp | Duzlu et al. 2016. |
|  |  | Fr1281R0.1 | GGACTAAGAGTACCTTTTTGAGT |  |  |  |
| *Ehrlichia* spp. and *Anaplasma* spp. | 16S rRNA | EHR16SD | GGTACCYACAGAAGAAGTCC | 95°C 2 min  40 cycles:  94°C 60 s  54°C 30 s  72°C 30 s  72°C – 5 min | 345 bp | Martin et al. 2005). |
|  |  | EHR16SR | TAGCACTCATCGTTTACAGC |  |  |  |
| *Theileria/Babesia* spp. | ssrRNA | Pirop-F | GTCTTGTAATTGGAATGATGG | 94°C 2 min  35 cycles:  94°C 30 s  50°C 30 s  72°C 60 s  72°C 7 min | 560 bp | Beck et al. 2009 |
|  |  | Pirop-R | CCAAAGACTTTGATTTCTCTC |  |  |  |
| *Trypanosoma spp.* |  | F | GAATATTAAACAATGCGCAG | 95°C 3 min  33 cycles:  94°C 30 s  52°C 30 s  72°C 30 s  72°C 10 min | 164 bp | Barghash et al. 2016. |
|  |  | R | CCATTTATTAGCTTTGTTGC |  |  |  |

**Table S2.** Microorganisms detected from ticks, host details and tick collection sites.

| Sample ID | Tick Species | Tick microorganisms’ description based on BLAST | Host species | Location |
| --- | --- | --- | --- | --- |
| 6f | *Hyalomma dromedarii* | *Francisella*-like endosymbiont of *Amblyomma paulopunctatum* isolate AmbpauTgab228 16S ribosomal RNA gene, partial sequence | Camel | Swehan, Al Ain |
| 16f | *Hyalomma dromedarii* | *Francisella*-like endosymbiont of *Amblyomma paulopunctatum* isolate AmbpauTgab228 16S ribosomal RNA gene, partial sequence | Camel | Nahil, Al Ain |
| 69f | *Hyalomma dromedarii* | *Francisella*-like endosymbiont of *Amblyomma paulopunctatum* isolate AmbpauTgab228 16S ribosomal RNA gene, partial sequence | Camel | Al Foah, Al Ain |
| 72f | *Hyalomma anatolicum* | *Candidatus* Midichloria mitochondrii isolate 13 16S ribosomal RNA gene, partial sequence | Goat | Emirates Park Zoo, Abu Dhabi |
| 72f | *Hyalomma anatolicum* | *Francisella*-like endosymbiont of *Hyalomma excavatum* isolate Hyaexc1 16S ribosomal RNA gene, partial sequence |  |  |
| 75f | *Hyalomma anatolicum* | *Candidatus* Midichloria mitochondrii isolate QtHaT5 16S ribosomal RNA gene, partial sequence | Goat | Emirates Park Zoo, Abu Dhabi |
| 75f | *Hyalomma anatolicum* | *Francisella*-like endosymbiont of *Hyalomma excavatum* isolate Hyaexc1 16S ribosomal RNA gene, partial sequence |  |  |
| 76f | *Hyalomma anatolicum* | *Candidatus* Midichloria mitochondrii isolate TK-A30 16S ribosomal RNA gene, partial sequence | Goat | Emirates Park Zoo, Abu Dhabi |
| 76f | *Hyalomma anatolicum* | *Francisella*-like endosymbiont of *Dermacentor marginatus* isolate F16TR-DmHp1 16S ribosomal RNA gene, partial sequence |  |  |
| 78f | *Hyalomma anatolicum* | *Candidatus* Midichloria mitochondrii isolate TK-A30 16S ribosomal RNA gene, partial sequence | Goat | Emirates Park Zoo, Abu Dhabi |
| 78f | *Hyalomma anatolicum* | *Francisella*-like endosymbiont of *Hyalomma excavatum* isolate Hyaexc1 16S ribosomal RNA gene, partial sequence |  |  |
| 79f | *Hyalomma anatolicum* | Candidatus Midichloria mitochondrii isolate TK-A36 16S ribosomal RNA gene, partial sequence | Sheep | Emirates Park Zoo, Abu Dhabi |
| 85f | *Hyalomma dromedarii* | *Francisella*-like endosymbiont of *Amblyomma paulopunctatum* isolate AmbpauTgab228 16S ribosomal RNA gene, partial sequence | Camel | Al Ain Central Market |
| 109f | *Hyalomma anatolicum* | *Candidatus* Midichloria mitochondrii isolate QtHaT5 16S ribosomal RNA gene, partial sequence | Sheep | Al Ain Central Market |
| 110f | *Hyalomma anatolicum* | *Candidatus* Midichloria mitochondrii isolate QtHaT5 16S ribosomal RNA gene, partial sequence | Cattle | Al Ain Central Market |
| 113f | *Hyalomma anatolicum* | *Candidatus* Midichloria mitochondrii isolate 13 16S ribosomal RNA gene, partial sequence | Cattle | Al Ain Central Market |
| 114f | *Hyalomma anatolicum* | *Candidatus* Midichloria mitochondrii isolate 37 16S ribosomal RNA gene, partial sequence | Cattle | Al Ain Central Market |
| 116f | *Hyalomma anatolicum* | Uncultured *Ehrlichia* sp. clone AR1-1 16S18S ribosomal RNA gene, partial sequence | Cattle | Al Ain Central Market |
| 116f | *Hyalomma anatolicum* | *Francisella*-like endosymbiont of *Hyalomma excavatum* isolate Hyaexc1 16S ribosomal RNA gene, partial sequence |  |  |
| 117m | *Hyalomma anatolicum* | *Candidatus* Midichloria mitochondrii isolate 13 16S ribosomal RNA gene, partial sequence | Cattle | Al Ain Central Market |
| 118f | *Hyalomma anatolicum* | Uncultured *Ehrlichia* sp. clone Ehr123 16S ribosomal RNA gene, partial sequence | Cattle | Al Ain Central Market |
| 119f | *Hyalomma anatolicum* | *Candidatus* Midichloria mitochondrii isolate QtHaT5 16S ribosomal RNA gene, partial sequence | Cattle | Al Ain Central Market |
| 133f | *Hyalomma dromedarii* | *Francisella*-like endosymbiont of *Amblyomma paulopunctatum* isolate AmbpauTgab228 16S ribosomal RNA gene, partial sequence | Camel | Sieh Al Hama, Al Ain |
| 154m | *Hyalomma anatolicum* | *Candidatus* Neoehrlichia sp. isolate IHU DO61S1 16S ribosomal RNA gene, partial sequence | Sheep | Ghnaymah, Al Ain |
| 155m | *Hyalomma anatolicum* | Uncultured *Ehrlichia* sp. clone AR1-1 16S18S ribosomal RNA gene, partial sequence | Cattle | Ghnaymah, Al Ain |
| 158f | *Hyalomma dromedarii* | *Francisella*-like endosymbiont of *Amblyomma paulopunctatum* isolate AmbpauTgab228 16S ribosomal RNA gene, partial sequence | Camel | Al Khazna abbatoir |
| 165f | *Hyalomma dromedarii* | *Francisella*-like endosymbiont of *Amblyomma paulopunctatum* isolate AmbpauTgab228 16S ribosomal RNA gene, partial sequence | Camel | Al Hiyar, Al Ain |
| 183f | *Hyalomma dromedarii* | *Candidatus* Midichloria mitochondrii isolate QtHaT5 16S ribosomal RNA gene, partial sequence | Camel | Al Hiyar, Al Ain |
| 218f | *Hyalomma dromedarii* | *Francisella*-like endosymbiont of *Amblyomma paulopunctatum* isolate AmbpauTgab228 16S ribosomal RNA gene, partial sequence | Camel | Al Rawdah, Al Ain |
| 235f | *Hyalomma dromedarii* | *Candidatus* Midichloria mitochondrii isolate 13 16S ribosomal RNA gene, partial sequence | Camel | Al Aflaj, Al Ain |
| 263f | *Hyalomma dromedarii* | *Francisella*-like endosymbiont of *Amblyomma paulopunctatum* isolate AmbpauTgab228 16S ribosomal RNA gene, partial sequence | Camel | Al Wagan, Abu Dhabi |
| 283f | *Hyalomma dromedarii* | *Francisella*-like endosymbiont of *Amblyomma paulopunctatum* isolate AmbpauTgab228 16S ribosomal RNA gene, partial sequence | Camel | Minhad, Dubai (1) |
| 288f | *Hyalomma dromedarii* | *Francisella*-like endosymbiont of *Amblyomma paulopunctatum* isolate AmbpauTgab228 16S ribosomal RNA gene, partial sequence | Camel | Minhad, Dubai (3) |
| 289f | *Hyalomma dromedarii* | *Francisella*-like endosymbiont of *Amblyomma paulopunctatum* isolate AmbpauTgab228 16S ribosomal RNA gene, partial sequence | Camel | Minhad, Dubai (4) |
| 303f | *Hyalomma dromedarii* | Francisella-like endosymbiont of Amblyomma paulopunctatum isolate AmbpauTgab228 16S ribosomal RNA gene, partial sequence | Camel | Al Mirfa, Al Dhafra |
| 319f | *Hyalomma dromedarii* | Francisella-like endosymbiont of Amblyomma paulopunctatum isolate AmbpauTgab228 16S ribosomal RNA gene, partial sequence | Camel | Madinat Zayed -1, Al Dhafra |
| 331f | *Hyalomma dromedarii* | *Francisella*-like endosymbiont of *Amblyomma paulopunctatum* isolate AmbpauTgab228 16S ribosomal RNA gene, partial sequence | Camel | Madinat Zayed-2, Al Dhafra |
| 343f | *Hyalomma dromedarii* | *Francisella*-like endosymbiont of *Amblyomma paulopunctatum* isolate AmbpauTgab228 16S ribosomal RNA gene, partial sequence | Camel | Madinat Zayed-3, Al Dhafra |
| 359f | *Hyalomma dromedarii* | *Francisella*-like endosymbiont of *Amblyomma paulopunctatum* isolate AmbpauTgab228 16S ribosomal RNA gene, partial sequence | Camel | Liwa-1, Al Dhafra |
| 366f | *Hyalomma dromedarii* | *Francisella*-like endosymbiont of *Amblyomma paulopunctatum* isolate AmbpauTgab228 16S ribosomal RNA gene, partial sequence | Camel | Liwa-2, Al Dhafra |
| 385f | *Hyalomma dromedarii* | *Francisella*-like endosymbiont of *Amblyomma paulopunctatum* isolate AmbpauTgab228 16S ribosomal RNA gene, partial sequence | Camel | Liwa-3, Al Dhafra |
| 392f | *Hyalomma dromedarii* | *Francisella*-like endosymbiont of *Amblyomma paulopunctatum* isolate AmbpauTgab228 16S ribosomal RNA gene, partial sequence | Camel | Liwa-4, Al Dhafra |
| 401f | *Hyalomma dromedarii* | *Francisella*-like endosymbiont of *Amblyomma paulopunctatum* isolate AmbpauTgab228 16S ribosomal RNA gene, partial sequence | Camel | Liwa-5, Al Dhafra |
| 410f | *Hyalomma dromedarii* | *Francisella*-like endosymbiont of *Amblyomma paulopunctatum* isolate AmbpauTgab228 16S ribosomal RNA gene, partial sequence | Camel | Ghayathi-1, Al Dhafra |
| 426f | *Hyalomma dromedarii* | *Francisella*-like endosymbiont of *Amblyomma paulopunctatum* isolate AmbpauTgab228 16S ribosomal RNA gene, partial sequence | Camel | Ghayathi-2, Al Dhafra |
| 442f | *Hyalomma dromedarii* | *Francisella*-like endosymbiont of *Amblyomma paulopunctatum* isolate AmbpauTgab228 16S ribosomal RNA gene, partial sequence | Camel | Ghayathi-3, Al Dhafra |
| 455f | *Hyalomma dromedarii* | *Francisella*-like endosymbiont of *Amblyomma paulopunctatum* isolate AmbpauTgab228 16S ribosomal RNA gene, partial sequence | Camel | Ghayathi-4, Al Dhafra |
| 470f | *Hyalomma dromedarii* | *Francisella*-like endosymbiont of *Amblyomma paulopunctatum* isolate AmbpauTgab228 16S ribosomal RNA gene, partial sequence | Camel | Al Sila-1, Al Dhafra |
| 484f | *Hyalomma dromedarii* | *Francisella*-like endosymbiont of *Amblyomma paulopunctatum* isolate AmbpauTgab228 16S ribosomal RNA gene, partial sequence | Camel | Al Sila-2, Al Dhafra |
| 498f | *Hyalomma dromedarii* | *Francisella*-like endosymbiont of *Amblyomma paulopunctatum* isolate AmbpauTgab228 16S ribosomal RNA gene, partial sequence | Camel | Al Sila-3, Al Dhafra |
| 514f | *Hyalomma dromedarii* | *Francisella*-like endosymbiont of *Amblyomma paulopunctatum* isolate AmbpauTgab228 16S ribosomal RNA gene, partial sequence | Camel | Jebel Mileina-1, Sharjah |
| 515f | *Hyalomma dromedarii* | *Francisella*-like endosymbiont of *Amblyomma paulopunctatum* isolate AmbpauTgab228 16S ribosomal RNA gene, partial sequence | Camel | Jebel Mileina-2, Sharjah |
| 520f | *Hyalomma dromedarii* | *Francisella*-like endosymbiont of *Amblyomma paulopunctatum* isolate AmbpauTgab228 16S ribosomal RNA gene, partial sequence | Camel | Jebel Mileina-3, Sharjah |
| 522f | *Hyalomma dromedarii* | *Francisella*-like endosymbiont of *Amblyomma paulopunctatum* isolate AmbpauTgab228 16S ribosomal RNA gene, partial sequence | Camel | Jebel Mileina-4, Sharjah |
| 536f | *Hyalomma dromedarii* | *Francisella*-like endosymbiont of *Amblyomma paulopunctatum* isolate AmbpauTgab228 16S ribosomal RNA gene, partial sequence | Camel | Al Digdaga, Ras Al-Khaimah |
| 542f | *Hyalomma dromedarii* | *Francisella*-like endosymbiont of *Amblyomma paulopunctatum* isolate AmbpauTgab228 16S ribosomal RNA gene, partial sequence | Camel | Al Morgbat, Umm Al Quwain |
| 547f | *Hyalomma dromedarii* | *Francisella*-like endosymbiont of *Amblyomma paulopunctatum* isolate AmbpauTgab228 16S ribosomal RNA gene, partial sequence | Camel | Falaj Al Sheikh, Umm Al Quwain |
| 552f | *Hyalomma dromedarii* | *Francisella*-like endosymbiont of *Amblyomma paulopunctatum* isolate AmbpauTgab228 16S ribosomal RNA gene, partial sequence | Camel | Bulaida, Fujairah |
| 555f | *Hyalomma dromedarii* | *Francisella*-like endosymbiont of *Amblyomma paulopunctatum* isolate AmbpauTgab228 16S ribosomal RNA gene, partial sequence | Camel | Al Tallah-1, Ajman |
| 560f | *Hyalomma dromedarii* | *Candidatus* Midichloria mitochondrii isolate QtHaT5 16S ribosomal RNA gene, partial sequence | Camel | Al Tallah-2, Ajman |
| 563f | *Hyalomma anatolicum* | Uncultured *Ehrlichia* sp. clone AR1-1 16S18S ribosomal RNA gene, partial sequence | Cattle | Dubai Cattle Market |
| 564f | *Hyalomma anatolicum* | *Candidatus* Midichloria mitochondrii isolate TK-A36 16S ribosomal RNA gene, partial sequence | Cattle | Dubai Cattle Market |
| 565f | *Hyalomma anatolicum* | Candidatus *Midichloria mitochondrii* isolate TK-A36 16S ribosomal RNA gene, partial sequence | Cattle | Dubai Cattle Market |
| 566f | *Hyalomma anatolicum* | *Candidatus* Midichloria mitochondrii isolate EV17 16S ribosomal RNA gene, partial sequence | Cattle | Dubai Cattle Market |
| 568f | *Hyalomma anatolicum* | *Candidatus* Midichloria mitochondrii isolate TK-A36 16S ribosomal RNA gene, partial sequence | Cattle | Dubai Cattle Market |
| 576f | *Hyalomma anatolicum* | *Candidatus* Midichloria mitochondrii isolate TK-A36 16S ribosomal RNA gene, partial sequence | Cattle | Dubai Cattle Market |
| 576f | *Hyalomma anatolicum* | *Francisella*-like endosymbiont of *Hyalomma excavatum* isolate Hyaexc1 16S ribosomal RNA gene, partial sequence | Cattle | Dubai Cattle Market |
| 579f | *Hyalomma anatolicum* | *Candidatus* Midichloria mitochondrii isolate TK-A36 16S ribosomal RNA gene, partial sequence | Cattle | Dubai Cattle Market |
| 584f | *Hyalomma anatolicum* | *Candidatus* Midichloria mitochondrii isolate QtHaT5 16S ribosomal RNA gene, partial sequence | Cattle | Dubai Cattle Market |
| 585f | *Hyalomma anatolicum* | *Candidatus* Midichloria mitochondrii isolate QtHaT5 16S ribosomal RNA gene, partial sequence | Cattle | Dubai Cattle Market |
| 586f | *Hyalomma anatolicum* | *Candidatus* Midichloria mitochondrii isolate QtHaT5 16S ribosomal RNA gene, partial sequence | Cattle | Dubai Cattle Market |
| 588f | *Hyalomma anatolicum* | *Candidatus* Midichloria mitochondrii isolate QtHaT5 16S ribosomal RNA gene, partial sequence | Cattle | Dubai Cattle Market |
| 594f | *Hyalomma anatolicum* | *Candidatus* Midichloria mitochondrii isolate TK-A36 16S ribosomal RNA gene, partial sequence | Cattle | Dubai Cattle Market |
| 599f | *Hyalomma anatolicum* | *Candidatus* Midichloria mitochondrii isolate QtHaT5 16S ribosomal RNA gene, partial sequence | Cattle | Dubai Cattle Market |
| 600f | *Hyalomma anatolicum* | *Candidatus* Midichloria mitochondrii isolate QtHaT5 16S ribosomal RNA gene, partial sequence | Cattle | Dubai Cattle Market |
| 601f | *Hyalomma anatolicum* | *Candidatus* Midichloria mitochondrii isolate 13 16S ribosomal RNA gene, partial sequence | Cattle | Dubai Cattle Market |
| 602f | *Hyalomma anatolicum* | *Candidatus* Midichloria mitochondrii isolate 13 16S ribosomal RNA gene, partial sequence | Cattle | Dubai Cattle Market |
| 604f | *Hyalomma anatolicum* | *Candidatus* Midichloria mitochondrii isolate 13 16S ribosomal RNA gene, partial sequence | Cattle | Dubai Cattle Market |
| 605f | *Hyalomma anatolicum* | *Candidatus* Midichloria mitochondrii isolate 13 16S ribosomal RNA gene, partial sequence | Cattle | Dubai Cattle Market |
| 607f | *Hyalomma anatolicum* | *Candidatus* Midichloria mitochondrii isolate QtHaT5 16S ribosomal RNA gene, partial sequence | Cattle | Dubai Cattle Market |
| 609f | *Hyalomma anatolicum* | *Candidatus* Midichloria mitochondrii isolate QtHaT5 16S ribosomal RNA gene, partial sequence | Cattle | Dubai Cattle Market |
| 614f | *Hyalomma anatolicum* | *Candidatus* Midichloria mitochondrii isolate QtHaT5 16S ribosomal RNA gene, partial sequence | Cattle | Dubai Cattle Market |
| 619f | *Hyalomma anatolicum* | *Candidatus* Midichloria mitochondrii isolate 13 16S ribosomal RNA gene, partial sequence | Cattle | Dubai Cattle Market |
| 620f | *Hyalomma anatolicum* | Uncultured *Ehrlichia* sp. clone VC2017ESPP72 16S ribosomal RNA gene, partial sequence | Cattle | Dubai Cattle Market |
| 622f | *Hyalomma anatolicum* | *Candidatus* Midichloria mitochondrii isolate 13 16S ribosomal RNA gene, partial sequence | Sheep | Dubai Cattle Market |
| 623f | *Hyalomma anatolicum* | *Candidatus* Midichloria mitochondrii isolate 13 16S ribosomal RNA gene, partial sequence | Sheep | Dubai Cattle Market |
| 623f | *Hyalomma anatolicum* | *Francisella*-like endosymbiont of *Dermacentor marginatus* isolate F16TR-DmHp1 16S ribosomal RNA gene, partial sequence |  |  |
| 625f | *Hyalomma anatolicum* | *Candidatus* Midichloria mitochondrii isolate QtHaT5 16S ribosomal RNA gene, partial sequence | Cattle | Sharjah Livestock Market |
| 626f | *Hyalomma anatolicum* | *Candidatus* Midichloria mitochondrii isolate TK-A30 16S ribosomal RNA gene, partial sequence | Cattle | Sharjah Livestock Market |
| 627f | *Hyalomma anatolicum* | *Candidatus* Midichloria mitochondrii isolate TK-A36 16S ribosomal RNA gene, partial sequence | Cattle | Sharjah Livetock Market |
| 629f | *Hyalomma anatolicum* | *Candidatus* Midichloria mitochondrii isolate TK-A36 16S ribosomal RNA gene, partial sequence | Cattle | Sharjah Livestock Market |
| 630f | *Hyalomma anatolicum* | *Candidatus* Midichloria mitochondrii isolate TK-A36 16S ribosomal RNA gene, partial sequence | Cattle | Sharjah Livestock Market |
| 631f | *Hyalomma anatolicum* | *Candidatus* Midichloria mitochondrii isolate TK-A36 16S ribosomal RNA gene, partial sequence | Cattle | Sharjah Livestock Market |
| 632f | *Hyalomma anatolicum* | Uncultured *Ehrlichia* sp. clone AR1-1 16S18S ribosomal RNA gene, partial sequence | Cattle | Sharjah Livestock Market |
| 633f | *Hyalomma anatolicum* | *Candidatus* Midichloria mitochondrii isolate TK-A36 16S ribosomal RNA gene, partial sequence | Cattle | Sharjah Livestock Market |
| 634f | *Hyalomma anatolicum* | *Candidatus* Midichloria mitochondrii isolate ZBSC1023 16S ribosomal RNA gene, partial sequence | Cattle | Sharjah Livestock Market |
| 635f | *Hyalomma anatolicum* | *Candidatus* Midichloria mitochondrii isolate ZBSC1023 16S ribosomal RNA gene, partial sequence | Cattle | Sharjah Livestock Market |
| 635f | *Hyalomma anatolicum* | *Francisella*-like endosymbiont of *Hyalomma excavatum* isolate Hyaexc1 16S ribosomal RNA gene, partial sequence |  |  |
| 636f | *Hyalomma anatolicum* | *Candidatus* Midichloria mitochondrii isolate TK-A36 16S ribosomal RNA gene, partial sequence | Cattle | Sharjah Livestock Market |
| 637f | *Hyalomma anatolicum* | *Candidatus* Midichloria mitochondrii isolate TK-A36 16S ribosomal RNA gene, partial sequence | Cattle | Sharjah Livestock Market |
| 637f | *Hyalomma anatolicum* | *Theileria annulata* small subunit ribosomal RNA gene, partial sequence |  |  |
| 638f | *Hyalomma anatolicum* | *Candidatus* Midichloria mitochondrii isolate ZBSC1023 16S ribosomal RNA gene, partial sequence | Cattle | Sharjah Livestock Market |
| 639f | *Hyalomma anatolicum* | *Candidatus* Midichloria mitochondrii isolate TK-A36 16S ribosomal RNA gene, partial sequence | Cattle | Sharjah Livestock Market |
| 640f | *Hyalomma anatolicum* | *Candidatus* Midichloria mitochondrii isolate TK-A36 16S ribosomal RNA gene, partial sequence | Cattle | Sharjah Livestock Market |
| 641f | *Hyalomma anatolicum* | *Candidatus* Midichloria mitochondrii isolate TK-A36 16S ribosomal RNA gene, partial sequence | Cattle | Sharjah Livestock Market |
| 642f | *Hyalomma anatolicum* | *Candidatus* Midichloria mitochondrii isolate QtHaT5 16S ribosomal RNA gene, partial sequence | Cattle | Sharjah Livestock Market |
| 643f | *Hyalomma anatolicum* | *Candidatus* Midichloria mitochondrii isolate TK-A36 16S ribosomal RNA gene, partial sequence | Cattle | Sharjah Livestock Market |
| 644f | *Hyalomma anatolicum* | *Candidatus* Midichloria mitochondrii isolate TK-A36 16S ribosomal RNA gene, partial sequence | Cattle | Sharjah Livestock Market |
| 644f | *Hyalomma anatolicum* | *Theileria annulata* small subunit ribosomal RNA gene, partial sequence |  |  |
| 645f | *Hyalomma anatolicum* | *Candidatus* Midichloria mitochondrii isolate TK-A36 16S ribosomal RNA gene, partial sequence | Cattle | Sharjah Livestock Market |
| 646f | *Hyalomma anatolicum* | *Candidatus* Midichloria mitochondrii isolate QtHaT5 16S ribosomal RNA gene, partial sequence | Cattle | Sharjah Livestock Market |
| 647f | *Hyalomma anatolicum* | *Candidatus* Midichloria mitochondrii isolate QtHaT5 16S ribosomal RNA gene, partial sequence | Cattle | Sharjah Livestock Market |
| 648f | *Hyalomma anatolicum* | *Candidatus* Midichloria mitochondrii isolate 13 16S ribosomal RNA gene, partial sequence | Cattle | Sharjah Livestock Market |
| 649f | *Hyalomma anatolicum* | *Candidatus* Midichloria mitochondrii isolate QtHaT5 16S ribosomal RNA gene, partial sequence | Cattle | Sharjah Livestock Market |
| 650f | *Hyalomma anatolicum* | *Candidatus* Midichloria mitochondrii isolate 13 16S ribosomal RNA gene, partial sequence | Cattle | Sharjah Livestock Market |
| 650f | *Hyalomma anatolicum* | *Theileria annulata* small subunit ribosomal RNA gene, partial sequence |  |  |
| 651m | *Hyalomma anatolicum* | Uncultured *Ehrlichia* sp. clone AR1-1 16S18S ribosomal RNA gene, partial sequence | Cattle | Sharjah Livestock Market |
| 652f | *Hyalomma anatolicum* | *Candidatus* Midichloria mitochondrii isolate QtHaT5 16S ribosomal RNA gene, partial sequence | Cattle | Sharjah Livestock Market |
| 653f | *Hyalomma anatolicum* | *Candidatus* Midichloria mitochondrii isolate TK-A36 16S ribosomal RNA gene, partial sequence | Cattle | Sharjah Livestock Market |
| 655f | *Hyalomma anatolicum* | Uncultured *Ehrlichia* sp. clone AR1-1 16S18S ribosomal RNA gene, partial sequence | Cattle | Sharjah Livestock Market |
| 657f | *Hyalomma anatolicum* | *Candidatus* Midichloria mitochondrii isolate QtHaT5 16S ribosomal RNA gene, partial sequence | Cattle | Sharjah Livestock Market |
| 658f | *Hyalomma anatolicum* | *Candidatus* Midichloria mitochondrii isolate QtHaT5 16S ribosomal RNA gene, partial sequence | Cattle | Sharjah Livestock Market |
| 659f | *Hyalomma anatolicum* | *Candidatus* Midichloria mitochondrii isolate QtHaT5 16S ribosomal RNA gene, partial sequence | Cattle | Sharjah Livestock Market |
| 660f | *Hyalomma anatolicum* | *Candidatus* Midichloria mitochondrii isolate QtHaT5 16S ribosomal RNA gene, partial sequence | Cattle | Sharjah Livestock Market |
| 662f | *Hyalomma anatolicum* | *Candidatus* Midichloria mitochondrii isolate TK-A36 16S ribosomal RNA gene, partial sequence | Cattle | Sharjah Livestock Market |
| 663f | *Hyalomma anatolicum* | *Candidatus* Midichloria mitochondrii isolate TK-A36 16S ribosomal RNA gene, partial sequence | Cattle | Sharjah Livestock Market |
| 663f | *Hyalomma anatolicum* | *Theileria annulata* small subunit ribosomal RNA gene, partial sequence |  |  |
| 664f | *Hyalomma anatolicum* | *Candidatus* Midichloria mitochondrii isolate TK-A36 16S ribosomal RNA gene, partial sequence | Cattle | Sharjah Livestock Market |
| 664f | *Hyalomma anatolicum* | *Theileria annulata* isolate IQ-camel No.6 small subunit ribosomal RNA gene, partial sequence |  |  |
| 665f | *Hyalomma anatolicum* | *Candidatus* Midichloria mitochondrii isolate QtHaT5 16S ribosomal RNA gene, partial sequence | Cattle | Sharjah Livestock Market |
| 670f | *Hyalomma anatolicum* | *Candidatus* Midichloria mitochondrii isolate QtHaT5 16S ribosomal RNA gene, partial sequence | Cattle | Sharjah Livestock Market |
| 672f | *Hyalomma anatolicum* | *Candidatus* Midichloria mitochondrii isolate TK-A30 16S ribosomal RNA gene, partial sequence | Cattle | Sharjah Livestock Market |
| 674f | *Hyalomma anatolicum* | *Candidatus* Midichloria mitochondrii isolate 13 16S ribosomal RNA gene, partial sequence | Cattle | Sharjah Livestock Market |
| 675f | *Hyalomma anatolicum* | *Candidatus* Midichloria mitochondrii isolate QtHaT5 16S ribosomal RNA gene, partial sequence | Cattle | Sharjah Livestock Market |
| 675f | *Hyalomma anatolicum* | *Theileria annulata* small subunit ribosomal RNA gene, partial sequence |  |  |
| 678f | *Hyalomma anatolicum* | *Candidatus* Midichloria mitochondrii isolate QtHaT5 16S ribosomal RNA gene, partial sequence | Cattle | Sharjah Livestock Market |

**Table S3.** Query sequence similarities with GenBank subject sequences.

| **Species** | **Accession ID**  **Current study** | **GenBank/Reference**  **Accession number** | **Similarity %** | **Country** |
| --- | --- | --- | --- | --- |
| *Theileria annulata* | | | | |
| *Theileria annulata* | PV954755 | EU083800 | 99.80 | China |
| *Theileria annulata* | PV955656 | AY508464 | 99.81 | Turkey |
| *Francisella*-like endosymbiont | | | | |
| *Francisella*-like endosymbiont | PV954819 | PQ460966 | 99.79 | Egypt |
| *Francisella*-like endosymbiont | PV954827 | MW287952 | 100 | France |
| *Francisella*-like endosymbiont | PV956212 | PP486356 | 100 | Turkey |
| *Francisella*-like endosymbiont | PV958095 | PQ460974 | 99.69 | Egypt |
| *Francisella*-like endosymbiont | PV959325 | PQ460972 | 99.79 | Egypt |
| *Francisella*-like endosymbiont | PV959455 | PQ275516 | 99.69 | Egypt |
| *Candidatus* Neoehrlichia sp. | | | | |
| *Candidatus Neoehrlichia sp.* | PV953365 | OM692212 | 100 | Algeria |
| Uncultured *Ehrlichia* sp. | | | | |
| Uncultured *Ehrlichia* sp. | PV955939 | OQ545726 | 99.65 | Pakistan |
| Uncultured *Ehrlichia* sp. | PV955725 | PQ896497 | 99.65 | China |
| *Ehrlichia* sp. | PV955650 | AF497581 | 100 | Thailand |
| Uncultured *Ehrlichia* sp. | PV954797 | ON527365 | 100 | Pakistan |
| *Candidatus* Midichloria mitochondrii | | | | |
| *Candidatus* Midichloria mitochondrii | PV952972 | PV696905 | 100 | Chad |
| *Candidatus* Midichloria mitochondrii | PV956164 | PV696904 | 100 | Chad |
| *Candidatus* Midichloria mitochondrii | PV952994 | MZ476204 | 100 | Morocco |
| *Candidatus* Midichloria mitochondrii | PV956136 | PV696904 | 100 | Chad |
| *Candidatus* Midichloria mitochondrii | PV952993 | MZ476204 | 100 | Morocco |
